# Biology of primary breast cancer in older women beyond routine biomarkers

**DOI:** 10.1007/s12282-021-01266-5

**Published:** 2021-06-24

**Authors:** R. M. Parks, L. H. Alfarsi, A. R. Green, K. L. Cheung

**Affiliations:** Nottingham Breast Cancer Research Centre, School of Medicine, Royal Derby Hospital Centre, University of Nottingham, Uttoxeter Road, Derby, DE22 3DT UK

**Keywords:** Biology, Primary operable breast cancer, Biomarker, Elderly, Older women

## Abstract

**Purpose:**

There are numerous biomarkers which may have potential predictive and prognostic significance in breast cancer. This is extremely important in older adults, who may opt for less aggressive therapy. This work outlines the literature on biological assessment outside of standard biomarkers (defined as ER, PgR, HER2, Ki67) in women ≥ 65 years with primary operable invasive breast cancer, to determine which additional biomarkers are relevant to outcome in older women.

**Methods:**

Medline and Embase databases were searched. Studies were eligible if included ≥ 50 patients aged ≥ 65 years; stratified results by age; measured a biomarker outside of standard assay and reported patient data.

**Results:**

A total of 12 studies were appraised involving 5000 patients, measuring 28 biomarkers. The studies were extremely varied in methodology and outcome but three themes emerged: 1. Differences in biomarker expression between younger and older women, indicating that breast cancer in older women is generally less aggressive compared to younger women; 2. Relationship of biomarker expression with survival, suggesting biomarkers which may exclusively predict response to primary treatment in older women; 3. Association of biomarker with chemotherapy, suggesting that older patients should not be declined chemotherapy based on age alone.

**Conclusion:**

There is evidence to support further investigation of B-cell lymphoma (BCL2), liver kinase (LK)B1, epidermal growth factor receptor (EGFR), cytoplasmic cyclin-E, mucin (MUC)1 and cytokeratins (CKs) as potential predictive or prognostic markers in older women with breast cancer undergoing surgery. Studies exploring these biomarkers in larger cohorts and in women undergoing non-operative therapies are required.

**Supplementary Information:**

The online version contains supplementary material available at 10.1007/s12282-021-01266-5.

## Introduction

The number of women aged ≥ 65 years living with breast cancer worldwide will quadruple by 2040 [1, 2]. Despite this, there are few guidelines for management of breast cancer specific to older women. The older population have unique geriatric issues, which may impact their decision making process when considering treatment options, for example, comorbidity, fitness for surgery, level of social activity^3^. Furthermore, curative treatment alone may not be the goal in mind for older women; quality of life and preservation of function may be more important [4].

Traditionally, the biomarkers oestrogen receptor (ER), progesterone receptor (PR), human epidermal growth factor receptor (HER)2 and more recently, Ki67 have been measured in routine clinical practice. Over time the study of breast cancer has revealed it’s heterogeneous nature, with a spectrum of many subtypes with distinct biological features [5]. This is even more pertinent in the older patient; the older population generally exhibit less aggressive features regardless of subtype [6–9].

Therefore, treatment plans based on routinely measured biomarkers and our current understanding of disease subtypes may no longer be adequate for older women. For example, even within the group of patients who have tumours which are ER-positive, outcome differs depending on degree of ER-positivity [7]. Due to competing causes of death, it is hypothesised that there are a group of patients who may never face mortality from the disease [10].

Current predictive and prognostic tools available on the market for example Oncotype DX [11], a genomic test and Adjuvant! Online [12], which uses standard clinico-pathological features, are focussed towards adjuvant rather than primary treatment and recruitment of older women in their conception is lacking [13]. These existing tools were developed from patients undergoing primary surgery following which they were used to determine prognosis and as such, potential benefit from adjuvant therapies. There is a need for prognostic tools unique to older women with operable invasive breast cancer, who might not necessarily receive primary surgery.

The aim of this work is to review the evidence base for biological markers outside of those routinely measured in clinical practice and avenues for future research.

## Materials and methods

A review protocol was developed based on the Preferred Reporting Items for Systematic Review and Meta-Analysis (PRISMA) statement [14] and using the support of a Senior Research Librarian from the University of Nottingham.

### Search strategy

The Medline and Embase databases were searched using OVID interface on the 25th January 2021. The importance of the unique biology of breast cancer in older women has become more understood in the last decade therefore, the search was limited to studies published within the past 10 years. Studies were restricted to those published in English language and with full-text available.

Titles and abstracts were searched using the following terms: (breast cancer) AND (older OR elderly OR geriatric OR aged) AND (primary OR operable OR non-metastatic OR surgery) AND (biology OR biomarker(s) OR marker(s) or subtype). Duplicate publications were excluded.

Articles were screened by two independent researchers (RP and LA) in two stages: screening of titles and abstracts followed by the retrieval and screening of full-text articles. Discrepancies were resolved by discussion.

Inclusion criteria were as follows:

- Female participants only,
- Inclusion of at least 50 female participants ≥ 65 years of age with primary operable invasive breast cancer,
- Stratification of results by age,
- Measurement of biomarker outside of standard assay (standard assay defined as ER, PgR), HER2 and Ki67; luminal A, luminal B, basal or triple negative, HER2-enriched, or synonymous descriptions) on breast tissue sample,
- Full-text articles only.

Studies were excluded if:

- Biology or breast cancer not discussed,
- Restricted access to study report/data/full-text,
- Review article, letter to the editor, editorial report, case report, conference abstract,
- Cell culture or animal study.

### Data extraction

All data were extracted directly from the study text. When available, the following variables were extracted: year of publication; country; definition of older women; number of women ≥ 65 years with primary invasive breast cancer included; type of breast cancer and treatment; tumour subtype if known; summary of how biomarker(s) measured; key findings.

### Critical appraisal

Methodological quality of papers was assessed using the Reporting Recommendations for Tumour Marker Prognostic Studies (REMARK) criteria [15] and in accordance with the PRISMA statement [14]. Since the included studies were extremely varied in methodology and context, meta-analysis was not performed.

## Results

**Fig. 1:**
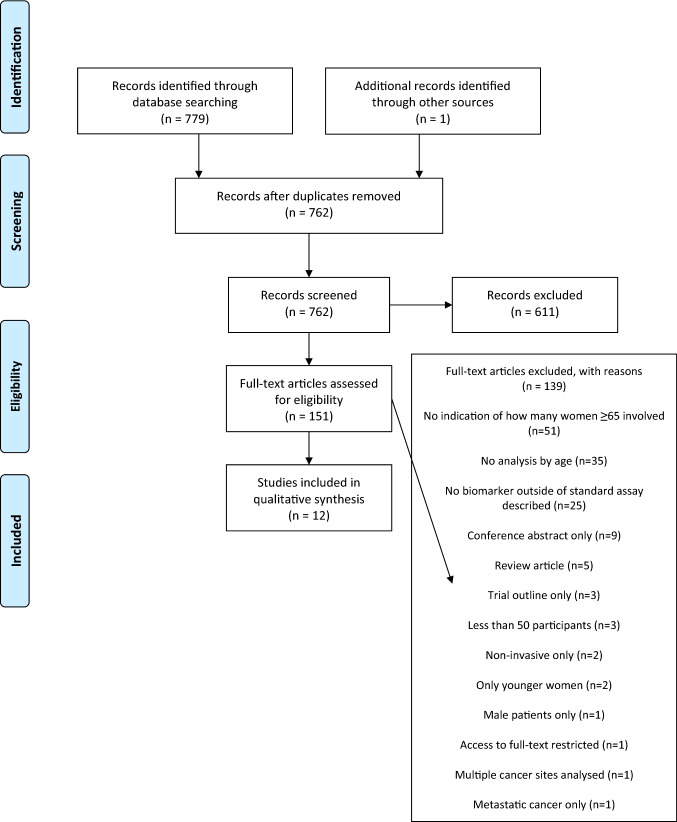
Flow chart demonstrating database search and study selection

Details of the literature search and study selection are shown in Fig. 1. Selected studies are referred to by the number allocated in Tables 1 and 2.

**Table 1:** Results of studies that directly compared an older to younger cohort of women

#	Author and date	Country	Age	*N*	Type of breast cancer	Breast cancer subtype	Rx	Biomarkers analysed	Method	Comparison of younger to older cohort	Other findings
1	Brouwers B, May 2015 [16]	Belgium	≥ 70	162	Stage I-III	Luminal A 61%; Luminal B 26%; HER2 5%; Triple negative 9%	All patients with GA before Rx	IL6, RANTES, MCP-1, IGF1	Blood, ELISA	IGF1, IL6, MCP1 associated with increasing age	IL6 correlated with clinical measure of frailty and could be used as a frailty biomarker
2	Syed BM, Jul 2014 [9]	UK	≥ 70	127	Stage I-III TNBC	Unknown, but all TNBC	Surgery	p53, CKs, BCL2, E-cadherin, MUC 1, EGFR	SE TMA, IHC	Older patients had lower levels of Ki67 and CK 7/8 and higher levels of BCL2 and CK18 compared to younger (*p* < 0.05)	Despite not having received adjuvant chemotherapy, the older series had clinical outcomes similar to younger patients. Positive EGFR status was associated with better BCSS (*p* = 0.01)
3	Syed BM, Mar 2013 [17]	UK	≥ 70	575	Stage I-III	Luminal A 38%; Luminal B 25%; All low expression/normal like 6%; Low ER luminal 15%; Basal-like 8%; HER2 8%	Surgery	p53, BCL2, MUC1, E-cadherin, CKs	SE TMA, IHC	Older women had higher expression of BCL2, MUC 1, BRCA1, BRCA2 and lower expression of p53, EGFR, CK17	A novel cluster (lower ER luminal) was identified, unique to the older cohort with different survival outcomes
4	Mieog JSD, Jan 2012 [44]	Netherlands	> 65	170	Stage I-III	Unknown, 72% ER + ve	Any surgical	ALDH1	SE TMA, IHC	Less likely to express ALDH1 with increasing age (*p* < 0.01)	ALDH1 expression not associated with any clinical outcome in older women

**Table 2:** Results of studies that reported results in an older cohort of women only

#	Author and date	Country	Age	N	Type of breast cancer	Breast cancer subtype	Rx	Biomarkers measured	Method	Findings
5	Parks RM, Nov 2020 [20]	UK	≥ 70	334	Stage I-III, ER + ve	Unknown, but all ER + ve tumours	PET	p53, cytokeratins, EGFR, BCL2, MUC1, VEGF, LKB1, BRCA1, HER3, HER4, PTEN, AIB1	CNB TMA, IHC	High BCL2 (*p* = 0.043) and low LKB1 (*p* = 0022) were associated with longer TTP; low MUC1 (0.021) was associated with better BCSS
6	Parks RM, July 2020 [21]	UK	≥ 70	693	Stage I-III	Consisted of luminal A, luminal B and low ER luminal (novel subtype); numbers of each unknown	Surgery *N* = 189 (35%), PET *N* = 333 (62%), U *N* = 14 (3%)	HER3, HER4, p53, EGFR, cytokeratins, MUC1, LKB1, BRCA1, BCL2, PTEN, VEGF, AIB1	CNB TMA, IHC	A novel cluster (lower ER luminal) was confirmed as previously identified in a previous study in SE TMAs in the same cohort
7	Lu G-W, June 2020 [22]	China	≥ 65	152	Stage I-III	Unknown, 72% of cohort ER + ve; HER2 + ve 7%	Mx	Translocator protein	Blood, ELISA	Post-op TP > 0.385 ng/mL increases risk of delirium and is associated with increased stage and grade (*p* < 0.001)
8	Johnston S Mar 2020 [23]	UK	≥ 70	517	Stage I-III	Unknown, 70.1% of cohort ER + ve; 91.7% HER2-ve	Any surgical	C-cyclin E	SE TMA, IHC	C-cyclin E was the only biological factor independently predictive of BCSS (HR = 6.23, CI 1.93–20.14, *p* = 0.002)
9	Syed BM, Jan 2019 [24]	UK	≥ 70	407	Stage I-III	Luminal A 36%; Luminal B 25%; Low ER luminal (novel cluster) 15%; All low expression / normal like 6%; Basal like 9%; HER2 + ve 9%	Any surgical	LKB1	SE TMA, IHC	Positive LKB1 expression is associated with better BCSS in patients receiving adjuvant endocrine therapy (*p* = 0.03)
10	Extermann M, Jan 2017 [25]	USA	≥ 65	56	Stage I-III, ER + ve,	Unknown, all ER + ve tumours	Surgery and adjuvant endocrine; adjuvant chemo *N* = 27	IL-6, d-dimer, albumin, IGF1, IGFBP3, TNF-alpha, vitamin D (markers of inflammation and frailty)	Blood, ELISA	There was no difference in expression of biomarkers in patients who completed chemotherapy compared to those who did not
11	Brouwers B, May 2016 [26]	Belgium	≥ 70	109	Any	Luminal A 40%; Luminal B 44%; Basal like 10%; HER2 + ve 6%	Any surgical; adjuvant chemo *N* = 57	Leucocyte telomere length, IL6, RANTES, MCP-1, IGF1, IL10 (markers of ageing and frailty)	Blood, qPCR and ELISA	Chemotherapy is unlikely to amplify clinical ageing or induce frailty at 1 year and does not increase mortality
12	Engels EC, Mar 2016 [27]	Netherlands	≥ 65	1698	Stage I-III	Unknown, 78% of cohort HER2-ve; ER + ve 72%	Surgical patients in FOCUS	PIK3CA	SE TMA, IHC	PIK3CA mutations had no impact on overall survival (*p* = 0.298)

### Summary

Twelve studies met the inclusion criteria and were included in this paper. These studies included a total of 5000 women aged ≥ 65 years with primary operable invasive breast cancer. All of the studies were observational with the exception of Study #10 and #11 which were case–control studies.

### Reporting standard

The results of REMARK assessment of full-text papers are given in Supplementary File 1. All papers achieved at least 75% of the REMARK criteria for reporting of a study including a biomarker. The most frequently missed criteria were number nine in relation to rationale of sample size (only two out of twelve studies included this) and 16 and 17 in the reporting of multivariate analyses and confidence intervals (only four and five studies included this information, respectively.

### General characteristics of the studies

The 12 studies included patients with primary breast cancer with a range of pathology. Details of tumour stage, grade, hormone receptor status and histological type where reported, are presented in Supplementary File 2.

Four studies made a direct comparison between measurement of biomarkers in an older compared to a younger cohort (Table 1) [9, 16–19]. The remaining eight studies measured a biomarker in an older cohort alone (Table 2) [20–27].

Six of the studies were performed in the UK [9, 17, 20, 21], four in other European countries [16, 18, 26, 27] and one each in China [22] and USA [25]. The number of eligible patients included per study varied enormously from 56 [25] to 1698 [27]. Eight studies defined older women as ≥ 70 years and five ≥ 65 years. All 12 studies examined patients treated by primary surgery with two of those also including those treated by non-operative therapies [20, 21].

A total of 28 biomarkers were measured in the 12 studies. The most frequently measured biomarkers outside of standard assay (as defined in the methodology) were tumour protein (p53), CKs, MUC1 and BCL2, each measured in four studies. Interleukin (IL)6, insulin growth factor (IGF)1, LKB1 and EGFR were measured in three studies. Regulated upon activation normal T cell expressed and presumably secreted (RANTES), monocyte chemotactic protein (MCP)1, phosphatase and tensin homolog (PTEN), amplified in breast cancer (AIB1), HER3, HER4, breast cancer gene (BRCA)1, vascular endothelial growth factor (VEGF) and e-cadherin appeared in two studies. The following biomarkers appears in one study each: IL10, PIK3CA, leucocyte telomere length (LTL), translocator protein (TP), cytoplasmic cyclin-E, d-dimer, albumin, IGF binding protein (BP)3, tumour necrosis factor (TNF)-alpha, vitamin D, alcohol dehydrogenase (ALDH)1. Only four studies (33%) gave a breakdown of tumours by classical subtypes.

Biomarker expression was based on tissue samples in eight studies; six on surgical excision (SE) specimen and two core needle biopsy (CNB). Four studies used peripheral blood samples.

### Significant findings

The following themes emerged: differences between older and younger women; relationship of biomarker with survival; association of biomarker with chemotherapy use.

#### Direct comparison of younger to older cohort

There was higher expression of IL6 [16], MCP1 [16], BCL2 [9], luminal cytokeratins [9, 20, 21], MUC1 [17], BRCA1 [17], BRCA2 [17], CEA [19] and CA 15-3 [19] in older women with primary breast cancer compared to younger women.

Lower levels of IGF1 [16], Ki67 [9], basal cytokeratins [17], p53 [17], EGFR [17] and ALDH1 [18] were seen in older women.

#### Relationship of biomarker with survival

The significant findings in relation to this theme are now summarised.

Study #3 identified a novel biological cluster (low ER luminal) present in older women undergoing surgery, which was not seen in younger women. The presence of this cluster was confirmed in core needle biopsy samples of older women in study #6. The low ER luminal cluster had a distinct biological pattern and had lower BCSS compared to luminal A and B type tumours.

A better BCSS was associated with low expression of MUC1 [21] and cytoplasmic cyclin-E [23] and high expression of EGFR [9].

High expression of BCL2 and low expression of LKB1 were associated with longer time to progression of disease in patients receiving primary endocrine therapy (PET) in study #5. Conversely in study #9, high expression of LKB1 was associated with better BCSS in patients receiving adjuvant endocrine therapy.

Expression of TP was associated with increased stage and grade of tumour at presentation in study #7. IL6 was associated with measures of clinical frailty [16].

#### Associations of biomarker with chemotherapy use

Despite not having received adjuvant chemotherapy, there was no difference in clinical outcome of older women with TNBC in study #2, compared to a series of younger women who did receive chemotherapy.

Study #10 found no difference in expression of biomarkers representing inflammation and frailty, in patients who completed chemotherapy compared to those who did not which was confirmed in study #11 which examined biomarkers of ageing and frailty.

## Discussion

The studies in this review add to the growing body of evidence that breast cancer in older women is less aggressive when compared to younger women and therefore, treatments offered should be tailored, instead of blanket treatment policies.

### Reporting standard

All papers achieved at least 75% of the REMARK criteria which is an acceptable rate. Many studies did not present information related to selection of sample size.

The main problem with studies of this kind is recruitment of many older women to these trials. Recruitment of older women to clinical studies is lacking due to bias by the clinician or patient, concerns regarding side effects of treatment and competing comorbidities. Therefore, a prescribed sample size may not be an appropriate goal in studies of older women, but simply to recruit as many participants as possible.

### Differences between older and younger women

IL6 and MCP1 are markers of ageing, therefore their increased expression in older women is anticipated as well as lower levels of IGF1, a marker of oxidative stress.

Higher expression of BRCA1 and 2 in older women could be explained by longer time to accumulate damage to these genes.

Many of our findings agree with the literature that breast cancer in older women generally displays less aggressive features for example, low expression of Ki67 (a marker of proliferation), ALDH (a breast cancer stem cell), EGFR (a growth factor receptor related to HER2) and p53 (a gene which promotes tumorigenesis) and high expression of BCL2 (an anti-apoptosis marker).

Contrary to this, are our findings related to MUC1 and cytokeratins.

MUC1 is an epithelial cell surface protein that is overexpressed in over 90% of breast as well as other cancers [28]. Evidence in breast cancers in younger women, show that high levels are associated with poor clinical outcome [29, 30]. We would therefore expect lower levels of MUC1 in older women, if they have less aggressive tumours.

In normal breast tissue, CK 5, CK14 and CK17 are expressed in myoepithelial cells and CK7, CK8, CK18, CK19, CK20 in ductal epithelium [31]. The role of expression of cytokeratins in breast cancer remains unclear. For example, high levels of CK18 have been shown to be associated with worse survival [32] as have CK17 and CK 5/6 [33]. A literature review by Haupt et al. [34] looked at the literature relating to basal-like breast carcinomas and confirmed that expression of basal CKs was an independent prognostic factor in lymph node negative cases, but in lymph node positive cases this was unclear. Overall, the evidence was suggestive that basal-like breast carcinomas are associated with worse clinical outcome. The contradictory results from this present literature review are certainly worthy of further investigation.

### Relationship of biomarker with survival

The novel biological cluster identified in study #3 and #6 (low ER luminal) had a distinct biological pattern and had lower breast cancer specific survival (BCSS) compared to luminal A and B type tumours. Low ER luminal had higher expression of CK7/8, BRCA1 and BCL-2, and lower expression of ER compared to Luminal A and B. Although the low ER luminal cluster has lower BCSS compared to luminal A and B response to surgery or PET within this cluster is similar. This could be interpreted as both treatments being equally as effective in this cluster, or that both treatments are equally unsatisfactory to treat this group of patients. This is an interesting finding and may suggest that in addition to conventional features such as grade, nodal status and ER status, cytokeratin expression may be significant in the role of response to PET.

A better BCSS was associated with low expression of MUC1 [21] and cytoplasmic cyclin-E [23] and high expression of EGFR [9].

Interestingly, in study #8, cytoplasmic cyclin E was the only independent biomarker of BCSS alongside a large panel of other biomarkers including EGFR There is a growing body of evidence to support its oncogenic role in various cancer types [35, 36]. Study #8 showed that at 10 years, 42% of older patients with cytoplasmic cyclin E-positive tumours had died of breast cancer versus 8% of negative cases (*p* < 0.0005). They concluded that older women with cytoplasmic cyclin E-negative tumours are unlikely to ever die of breast cancer. This information could significantly change the focus of primary treatment for breast cancer in older women, especially in comorbid patients.

Specifically in older women with TNBC in study #2 [9], EGFR has been shown to be more common in younger compared to older women. High expression of EGFR is known to correlate with poor clinical outcome in breast cancer [37]. There are now EGFR inhibitors on the market developed for use in other cancer types including lung and pancreatic cancer [28]. For breast cancer, EGFR inhibitors have not yet been successful in universally producing good response rates [38, 39].

High expression of BCL2 and low expression of LKB1 were associated with longer time to progression of disease in study #5. Conversely, in study #9 high expression of LKB1 was associated with better BCSS in patients-receiving adjuvant endocrine therapy, but not those undergoing surgery alone. Other studies to date examining LKB1 in are mainly cell line studies [40, 41]. LKB1 could be a potential therapeutic target.

BCL2 was found in higher proportions in breast cancers of older women in study numbers #2 and #3. BCL2 regulates cell death by either inhibiting or inducing apoptosis, therefore, positivity is associated with improved outcomes. This agrees with findings from a large biological characterisation study in 2003 by Daidone et al. [42] of over 14,000 primary breast cancers.

Expression of TP, a marker of inflammation, was associated with increased stage and grade of tumour in postoperative patients only; levels were undetectable pre-operatively [22]. Therefore, it is difficult to elicit how much of this is due simply to the process of surgical treatment.

IL6 was associated with chronological age as well as with measures of clinical frailty [16] as per the Balducci score. The authors suggested that IL6 could be used as a predictive marker to determine the toxicity of certain breast cancer treatments and may be a precursor to full geriatric assessment, a strategy that is recommended in the field of geriatric oncology [43].

### Associations of biomarker with chemotherapy use

In study #11 older women after surgery were assigned to either chemotherapy or a control group. The included biomarkers that represent ageing suggested a minor biological effect in the group receiving chemotherapy, but this was not clinically relevant. Therefore, the authors suggested that older breast cancer patients should not be denied chemotherapy based on age. However, in study #2 of patients with TNBC, there was no difference in survival outcome between older and younger patients, despite the older patients rarely receiving chemotherapy.

Study #10 did not find any differences in markers of inflammation, oxidation or frailty amongst patients receiving chemotherapy. Lower levels of Vitamin D were reported in patients who were treated by chemotherapy, however, there was little impact on measures of functional outcome at 2 years’ post-diagnosis. They suggested that supplementation of Vitamin D levels by exercise and/or oral supplements may be useful in these patients.

### Limitations

Comparability of studies is limited due to such a varied selection of studies with different methodology and aims. Although the papers described are important in their own merit as they contribute to the evidence based in older women, their heterogeneity means it is extremely difficult to perform a sound systematic review of the literature on this subject. Further work to examine each of three themes that emerged in this present review in more detail might be one way to overcome this. These themes are: comparison of biology between younger and women; relationship of biomarker with survival specific to older women; relationship of biomarker with chemotherapy use specific to older women.

Only 4 of the 12 studies highlighted in this paper breakdown included patients by tumour subtype. Even within these 4 papers, the definitions of tumour subtypes varied. Therefore, it was not possible to draw any firm conclusions regarding correlation between novel biomarkers reported in this paper and tumour subtypes.

Studies which have included the largest number of patients, are retrospective in nature. One of the main hurdles of biological studies is availability of patient tissue and blood samples and long-term follow-up data, which is often available in retrospective datasets. With updated ethical and legal guidance regarding handling of human tissue and consent, new prospective studies of this kind could face difficulty.

The majority of studies examining patient tissue, used surgical excision specimens to do so. This excludes the important group of older women who have non-surgical treatment and creates potential bias towards ER-negative tumours (who are not suitable for PET). Therefore, results may not be necessarily applicable to all older women with breast cancer.

Four studies did not examine patient tissue but instead used peripheral blood sampling. The methodology behind tissue versus blood sampling is clearly different, as is the range of biomarkers that can be measured by each method. Due to the small number of studies included in this present review we have considered all studies irrespective of sample method; however, the most significant findings have been found in those studies using tissue material for research alone.

Due to the small number of studies included in this review and in some of the individual studies, a small number of patients, no attempt was made to stratify patients by different treatments or individual pathological subgroups.

## Conclusions

There is an evidence base for the importance of biomarkers outside those currently measured in clinical practice, in breast cancer in older women, compared to younger women. However, the range of biomarkers and methodology used varies to such an extent it is difficult to form robust conclusions.

The biomarkers with the most evidence as highlighted in this paper which may have prognostic implications on breast cancer in older women include and are under reported in the current literature include: BCL2, cyclin E, EGFR, LKB1, MUC1 and CKs. Breast cancer in older women should not be treated the same as in their younger counterparts; there is a need for individualised treatment plans not purely based on chronological age.

Further measurement of these biomarkers using standardised methodology in larger numbers of older women will enable firm conclusions to be drawn about their predictive and prognostic value, primarily in a surgical setting. Biomarker assessment on a similar scale in CNB is urgently needed to address the same issues in those patients having non-operative therapies.

In the future, incorporation of these biomarkers into routine clinical practice may revolutionise the management of breast cancer in older women, alongside conventional factors.

## Supplementary Information

Below is the link to the electronic supplementary material.

Supplementary file1 Supplementary File 1: Results of REMARK assessment of full-text papers included (DOCX 20 kb)

Supplementary file2 Supplementary File 2: Additional pathological information of patients included in individual studies (DOCX 22 kb)

## Abbreviations

+ ve: Positive
− ve: Negative
AIB: Amplified in breast cancer
ALDH: Aldehyde dehydrogenase
AR: Androgen receptor
BCL: B-cell lymphoma
BCS: Breast conserving surgery
BCSS: Breast cancer specific survival
BRCA: Breast cancer gene
CA: Conference abstract
CDH: Chromodomain helicase DNA binding protein
CEA: Carcinoembryonic antigen
CK: Cytokeratin
EGFR: Epidermal growth factor receptor
ER: Oestrogen receptor
FP: Full paper
IGF: Insulin-like growth factor
IL: Interleukin
HER: Human epidermal growth factor receptor
HRD: Homologous recombination deficiency
KMT2C: Lysine methytransferase
LK: Liver kinase
MAP2K: Mitogen-activated protein kinase
MCP: Methyl-accepting chemotaxis protein
Mx: Mastectomy
MUC: Mucin
N: Number
NAHT: Neoadjuvant hormonal therapy
NCOR: Nuclear receptor corepressor
NF: Normal function
PAI: Plasminogen activator inhibitor
PgR: Progesterone receptor
PFS: Progression free survival
PTEN: Phosphatase and tensin homolog
RANTES: Regulated upon activation, normal T cell expressions and presumably secreted
RS: Recurrence score
RUNX: Runt-related transcription factor
TNF: Tumour necrosis factor
TP: Translocator protein
TP53: Tumour suppressor gene p53
TYPMS: Thymidine phosphorylase
U: Unknown
VEGF: Vascular endothelial growth factor
