# Supplementary material for: Biology of primary breast cancer in older women beyond routine biomarkers
Source: Breast Cancer. 2021 Jun 24;28(5):991–1001. doi: 10.1007/s12282-021-01266-5 (PMC8354915; doi:10.1007/s12282-021-01266-5)
Supplement: Supplementary file 1 — Supplementary file1 Supplementary File 1: Results of REMARK assessment of full-text papers included (DOCX 20 kb) [file 12282_2021_1266_MOESM1_ESM.docx]

| **#** | **Author and date** | **Retrospective or prospective** | **Tumour size** | **LN status** | **Grade** | **ER status** | **PR status** | **HER2 status** | **Histological Type** |
| --- | --- | --- | --- | --- | --- | --- | --- | --- | --- |
| 1 | Brouwers B,  May 2015 | Retrospective | T1 N=55  (28.1%), T2 N=79 (54.9%), T3 N=8 (5.6%), T4 N=2 (1.4%), | N0 N=83 (57.6%), N1 N=42 (29.2%), N2 N=10 (6.9%), N3 N=8 (5.6%), Nx N=1 (0.7%) | I 15.4% II 47.5% III 36.4% U 0.6% | - | - | - | Ductal N=111 (68.5%),  Lobular N=26 (15.4%), Ductal + lobular N=2 (1.2%), Ductal + other N=5 (3.1%), Other N=19 (11.7%) |
| 2 | Syed BM,  Jul 2014 | Retrospective | ≤2cm N=39 (33.3), >2cm N=78 (66.7%), | -ve N =50 (61%), 1=3 +ve nodes 20 (24.4%), ≥4 +ve nodes N=12 (14.6%) | I N=3 (2.8%), II N=19 (17.4%), III N=87 (79.8%) | All negative | All negative | All negative | Ductal N=107 (85.6%), Tubular N=4 (3.2%), Lobular N=5(4.0%), Other N=9 (7.2%) |
| 3 | Syed BM,  Mar 2013 | Retrospective | ≤3cm N=424 (81.5%),  >3cm N=96 (18.5%) | N0-1 | I/II N=253 III N=224 | +ve N=401 (70.1%), -ve N=171 (29.9%) | +ve N=317 (55.5%), -ve N=254 (44.5%) | +ve N=45 (7.5%),  -ve N=538 (92.3%) | - |
| 4 | Mieog JSD, Jan 2012 | Retrospective | T1 N=51 (31.3%),  T2 N=85 (52.1%),  T3/4 N=27 (16.6%) | -ve N=92 (57.5),  +ve N=68 (42.5%) | I N=24 (14.5%),  II N=82 (49.7%),  III N=59 (35.8%) | +ve N=117 (71.8%), -ve N=46 (28.2%) | +ve N=100 (62.1%), -ve N=61 (37.9%) | +ve N=9 (6.7%),  -ve N=125 (93.3%) | Ductal N=145 (87.9%),  Lobular N=20 (12.1%) |
| 5 | Parks RM, Nov 2020 | Retrospective | ≤2cm N=68 (33%), 2-5cm N=140(67%) | N0-1 | I N=39 (19%),  II N=144 (69%),  III 25 (12%) | All positive | - | - | - |
| 6 | Parks RM, July 2020 | Retrospective | 0.1-2cm N=122 (23%),  2.1-5cm N=163 (30%),  U N=252 (47%) | N0-1 | I N=62 (12%),  II N=206 (39%),  III N=30 (6%) | - | - | - | - |
| 7 | Lu G-W,  June 2020 | Prospective | <2cm N=80 (52.6%), ≥2cm N=72 (47.4%) | -ve N=69 (45.4%), +ve N=83 (54.6%) | I/II N=77 (50.7%), III N=75 (49.3%) | +ve N=85 (55.9%),  -ve N=67 (44.1%) | +ve N=75 (49.3%),  -ve N=77 (50.7%) | - | - |
| 8 | Johnston S  Mar 2020 | Retrospective | <20mm N=206 (39.8%),  ≥20mm N=329 (68.3%) | Stage I N=183 (57.5%),  Stage II N=98 (30.8%);  Stage III N=37 (11.6%) | I N=51 (12.0%),  II N=169 (39.8%);  III N=205 (48.2%) | +ve N=335 (70.1%),  -ve N=143 (29.9%) | - | +ve N=41 (8.3%). -ve N=453 (91.7%) | - |
| 9 | Syed BM,  Jan 2019 | Retrospective | <3cm 81.7%, | Among LKB1 +ve:  -ve 54.2% +ve 45.8% | Among LKB1 +ve:  I/II 44.5%,  III 55.5% | Among LKB1 +ve: +ve 69% -ve 31 % | Among LKB1 +ve: +ve 55.1%  -ve 48.9% | Among LKB1 +ve: +ve 9.6% -ve 90.4% | - |
| 10 | Extermann M,  Jan 2017 | Prospective | - | - | - | - | - | - | - |
| 11 | Brouwers B,  May 2016 | Prospective | T1 N=32 (29.4%),  T2 N=67 (61.5%), T3 N=6 (5.5%),  T4 N=4 (3.7%) | N0 N=45 (42.9%),  N1-3 N=60 (57.1%) | - | - | - | +ve N=6 (5.5%)  -ve N=103 (94.5%) | - |
| 12 | Engels EC,  Mar 2016 | Retrospective | - | - | I N=586, (37.1%)  II N=525 (33.2%),  III N=468 (29.6%) | ER and PR:  +ve N=1224 (82.1%),  -ve N=267 (17.9%) | - | +ve N=372 (21.9%),  -ve N=1326 (78.1%) | Ductal N=1278 (85.3%), Lobular N=179 (11.9%),  U N=42 (2.8%) |
