# Supplementary material for: Biology of primary breast cancer in older women beyond routine biomarkers
Source: Breast Cancer. 2021 Jun 24;28(5):991–1001. doi: 10.1007/s12282-021-01266-5 (PMC8354915; doi:10.1007/s12282-021-01266-5)
Supplement: Supplementary file 2 — Supplementary file2 Supplementary File 2: Additional pathological information of patients included in individual studies (DOCX 22 kb) [file 12282_2021_1266_MOESM2_ESM.docx]

**Supplementary File 1:** Results of REMARK assessment of full-text papers included

| **#** | **Author** | **1** | **2** | **3** | **4** | **5** | **6** | **7** | **8** | **9** | **10** | **11** | **12** | **13** | **14** | **15** | **16** | **17** | **18** | **19** | **20** | **Total** |
| --- | --- | --- | --- | --- | --- | --- | --- | --- | --- | --- | --- | --- | --- | --- | --- | --- | --- | --- | --- | --- | --- | --- |
| 1 | Brouwers B | Y | Y | Y | Y | Y | Y | Y | Y | X | Y | X | Y | Y | Y | Y | X | Y | Y | Y | Y | 17 |
| 2 | Syed BM | Y | Y | Y | Y | Y | Y | Y | Y | X | Y | Y | Y | Y | Y | Y | X | X | Y | X | Y | 16 |
| 3 | Syed BM | Y | Y | Y | Y | Y | Y | Y | Y | X | Y | Y | Y | Y | Y | Y | X | X | Y | Y | Y | 17 |
| 4 | Mieog JSD | Y | Y | Y | Y | Y | Y | Y | Y | Y | Y | Y | Y | Y | Y | Y | Y | Y | Y | Y | Y | 20 |
| 5 | Parks RM | Y | Y | Y | Y | Y | Y | Y | Y | X | Y | Y | Y | Y | X | Y | X | X | Y | Y | Y | 16 |
| 6 | Parks RM | Y | Y | Y | Y | Y | Y | Y | Y | X | Y | Y | Y | Y | X | Y | X | X | Y | Y | Y | 16 |
| 7 | Lu G-W | Y | Y | Y | Y | X | Y | X | Y | X | Y | Y | Y | Y | Y | Y | Y | Y | Y | X | Y | 16 |
| 8 | Johnston S | Y | Y | Y | Y | Y | Y | Y | Y | X | Y | Y | Y | Y | Y | Y | Y | X | Y | Y | Y | 18 |
| 9 | Syed BM | Y | Y | Y | Y | Y | Y | Y | Y | X | Y | Y | Y | Y | Y | Y | X | X | Y | Y | Y | 17 |
| 10 | Extermann M | Y | Y | Y | Y | Y | Y | Y | Y | Y | Y | X | Y | Y | X | Y | X | X | Y | Y | Y | 16 |
| 11 | Brouwers B | Y | Y | Y | Y | Y | X | Y | Y | X | Y | X | Y | Y | X | Y | X | Y | Y | Y | Y | 15 |
| 12 | Engels EC | Y | Y | Y | Y | Y | Y | Y | Y | X | Y | Y | Y | Y | Y | Y | Y | Y | Y | Y | Y | 19 |

(Y = criteria met; X = criteria not met)
